# Consecutive daily administration of intratracheal surfactant and human umbilical cord-derived mesenchymal stem cells attenuates hyperoxia-induced lung injury in neonatal rats

**DOI:** 10.1186/s13287-021-02335-4

**Published:** 2021-05-01

**Authors:** Hsiu-Chu Chou, Chien-Hsiang Chang, Chien-Han Chen, Willie Lin, Chung-Ming Chen

**Affiliations:** 1 Department of Anatomy and Cell Biology, School of Medicine, College of Medicine, Taipei Medical University, Taipei, Taiwan; 2 Department of Chemical Engineering, National Cheng Kung University, Tainan, Taiwan; 3 Meridigen Biotech Co., Ltd., Taipei, Taiwan; 4 Department of Pediatrics, Taipei Medical University Hospital, Taipei, Taiwan; 5 Department of Pediatrics, School of Medicine, College of Medicine, Taipei Medical University, Taipei, Taiwan

**Keywords:** Hyperoxia, Surfactant, Human umbilical cord-derived mesenchymal stem cells, Mean linear intercept, Vascular endothelial growth factor

## Abstract

**Background:**

Surfactant therapy is a standard of care for preterm infants with respiratory distress and reduces the incidence of death and bronchopulmonary dysplasia in these patients. Our previous study found that mesenchymal stem cells (MSCs) attenuated hyperoxia-induced lung injury and the combination therapy of surfactant and human umbilical cord-derived MSCs (hUC-MSCs) did not have additive effects on hyperoxia-induced lung injury in neonatal rats. The aim is to evaluate the effects of 2 consecutive days of intratracheal administration of surfactant and hUC-MSCs on hyperoxia-induced lung injury.

**Methods:**

Neonatal Sprague Dawley rats were reared in either room air (RA) or hyperoxia (85% O_2_) from postnatal days 1 to 14. On postnatal day 4, the rats received intratracheal injections of either 20 μL of normal saline (NS) or 20 μL of surfactant. On postnatal day 5, the rats reared in RA received intratracheal NS, and the rats reared in O_2_ received intratracheal NS or hUC-MSCs (3 × 10^4^ or 3 × 10^5^ cells). Six study groups were examined: RA + NS + NS, RA + surfactant + NS, O_2_ + NS + NS, O_2_ + surfactant + NS, O_2_ + surfactant + hUC-MSCs (3 × 10^4^ cells), and O_2_ + surfactant + hUC-MSCs (3 × 10^5^ cells). The lungs were excised for histological, western blot, and cytokine analyses.

**Results:**

The rats reared in hyperoxia and treated with NS yielded significantly higher mean linear intercepts (MLIs) and interleukin (IL)-1β and IL-6 levels and significantly lower vascular endothelial growth factors (VEGFs), platelet-derived growth factor protein expression, and vascular density than did those reared in RA and treated with NS or surfactant. The lowered MLIs and cytokines and the increased VEGF expression and vascular density indicated that the surfactant and surfactant + hUC-MSCs (3 × 10^4^ cells) treatment attenuated hyperoxia-induced lung injury. The surfactant + hUC-MSCs (3 × 10^5^ cells) group exhibited a significantly lower MLI and significantly higher VEGF expression and vascular density than the surfactant + hUC-MSCs (3 × 10^4^ cells) group did.

**Conclusions:**

Consecutive daily administration of intratracheal surfactant and hUC-MSCs can be an effective regimen for treating hyperoxia-induced lung injury in neonates.

## Background

Supraphysiological oxygen is often required to treat newborns with respiratory disorders. However, administering supplemental oxygen to newborn infants with respiratory failure can lead to lung injury. Term-born rat models are appropriate for studying the effects of hyperoxia on preterm infants with respiratory distress because rats are born at the saccular stage, which is approximately equivalent to a human gestational age of 30 weeks [1]. The prolonged exposure of neonatal rats to hyperoxia results in a decrease in alveolarization and vascularization similar to human bronchopulmonary dysplasia (BPD) [2, 3]. The pathogenesis of BPD is multifactorial, and oxygen toxicity plays a crucial role in the process of lung injury leading to BPD [4, 5].

Surfactant therapy is a standard of care for preterm infants with respiratory distress syndrome and can reduce the incidence of death and BPD [6]. Mesenchymal stem cells (MSCs) are multipotent stromal cells that have immunomodulatory, anti-inflammatory, and regenerative properties and have been demonstrated to treat hyperoxia-induced lung injury in newborn animals [7–14]. In previous study, we demonstrated that the addition of surfactant reduced the in vitro viability of human umbilical cord-derived MSCs (hUC-MSCs) through mitochondrial dysfunction and that a combination therapy of surfactant and hUC-MSCs had no additive effects on lung development in neonatal rats exposed to hyperoxia [14]. Early surfactant treatment was more effective in reducing mortality, air leak, BPD, and BPD or death compared with delayed surfactant treatment in preterm infants [15]. Animal study revealed that the early rather than late administration of intratracheal MSCs improved hyperoxia-induced lung injury in newborn rats [16]. However, the optimal time interval between surfactant and MSC administration for preterm infants remains unknown. The aim of this study is to evaluate the effects of 2 consecutive days of intratracheal administration of surfactant and hUC-MSCs on hyperoxia-induced lung injury. We hypothesized that consecutive daily administration of intratracheal surfactant and hUC-MSCs improves lung development and that high doses of hUC-MSCs enhance this effect on experimental BPD in neonatal rats. The aim of this study was to investigate the effects of consecutive daily administration of an animal-derived surfactant (Survanta) and hUC-MSCs on hyperoxia-induced lung injury in neonatal rats.

## Methods

### Isolation of human umbilical cord-derived mesenchymal stem cells

**Fig. 1:**
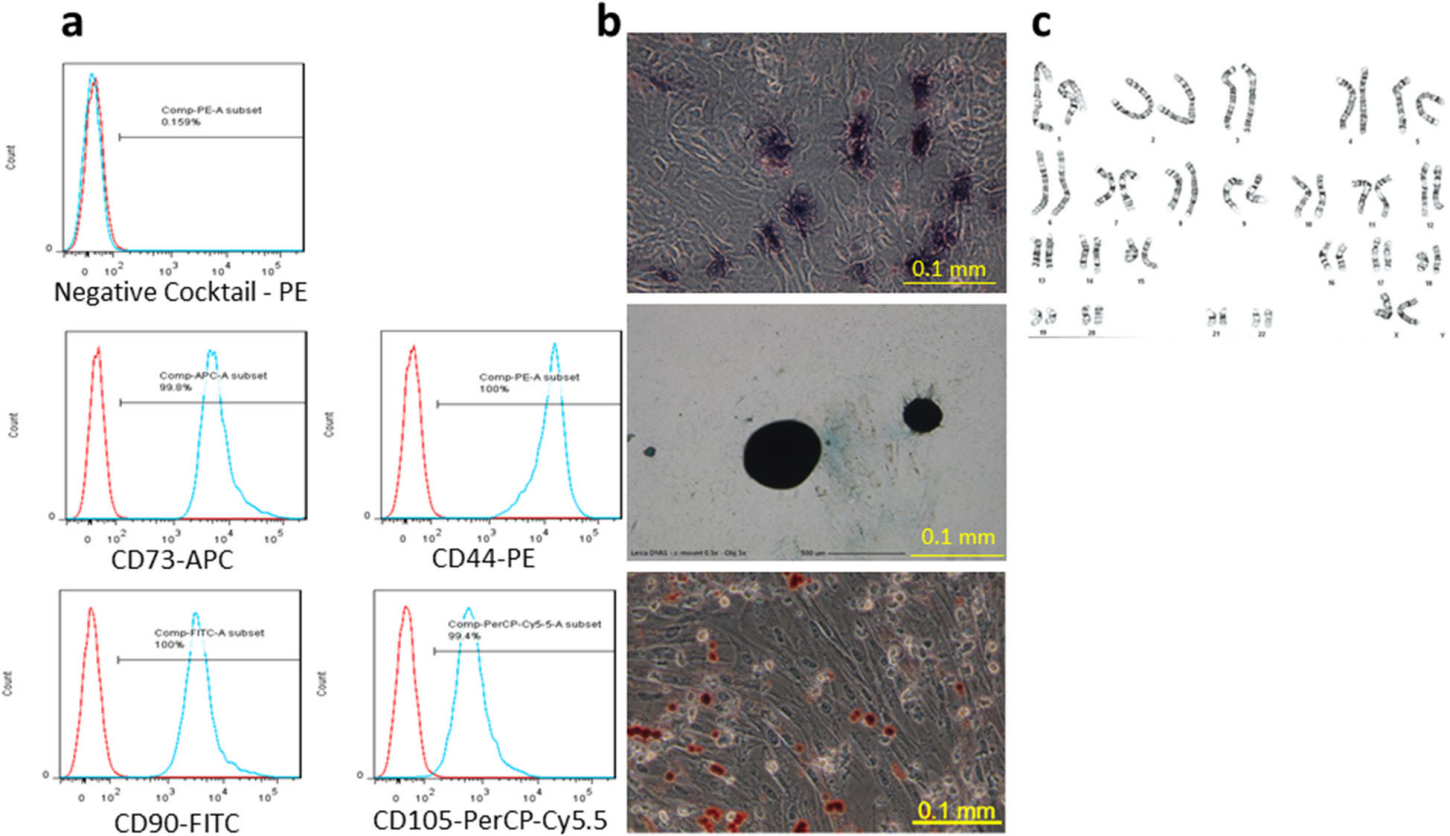
Characterization of hUC-MSCs. **a** The expression of human MSC-specific CD markers and **b** tri-lineage differentiation (from upper to lower: osteocyte, chondrocyte, and adipocyte) was performed to show the differentiation potency of hUC-MSCs. Differentiated cells were stained with the classical dyes: alkaline phosphatase for osteoblasts, alcian blue for chondroblasts, and Oil Red O for adipocytes. **c** The karyotype of hUC-MSCs was analyzed to ensure the chromosome stability

Human umbilical cord-derived mesenchymal stem cells were obtained from Meridigen Biotech Co., Ltd. (Taipei, Taiwan). The cells used in the present study were followed by the International Society for Cellular Therapy Guidelines. The umbilical cord tissue was collected under sterile conditions and digested with collagenase (SERVA, Heidelberg, Germany) for 120 min in a 37 °C incubator. Digestion was terminated in α-minimal essential culture medium (Invitrogen, Waltham, MA, USA) supplemented with 18% fetal bovine serum (Invitrogen), 4 ng/ml basic fibroblast growth factor (Peprotech, Rocky Hill, NJ, USA), and 50 mg/ml gentamicin. The cells were subsequently incubated in a humidified incubator with 5% CO_2_ at 37 °C for 3 days, at which point the culture medium was replenished, and the non-adherent cells were removed. hUC-MSCs were passaged once reached 80–90% confluence to the fourth generation. The hUC-MSCs were characterized by analyzing the expression of CD markers (CD44, CD73, CD90, and CD105) and the human leukocyte antigen-antigen D-related complex through flow cytometry (BD Stemflow™ hMSC Analysis Kit, BD, Franklin Lakes, NJ, USA) (Fig. 1a). The capability of trilineage differentiation (osteocytes, chondrocytes, and adipocytes) and the karyotyping result were examined, which revealed positive results (Fig. 1b). For long-term storage, hUC-MSCs were suspended in CryoStor CS10 (STEMCELL Technologies, Vancouver, BC, Canada) and cryopreserved in a vapor phase liquid nitrogen tank. This study was approved by the Ethics Committee of the National Cheng Kung University Hospital Institutional Review Board (Tainan, Taiwan). All subjects received written and oral information prior to inclusion and provided informed consent. All study processes were carried out in accordance with the approved study protocol (IRB No.: A-BR-104-045). The hUC-MSCs, in a cryovial, were thawed in a 37 °C water bath for 2 min, and the cell concentration of hUC-MSCs was prepared by dilution with clinical grade normal saline (NS) into 1.5 × 10^6^ or 1.5 × 10^7^ cells/mL.

### Animal model and experimental groups

**Fig. 2:**
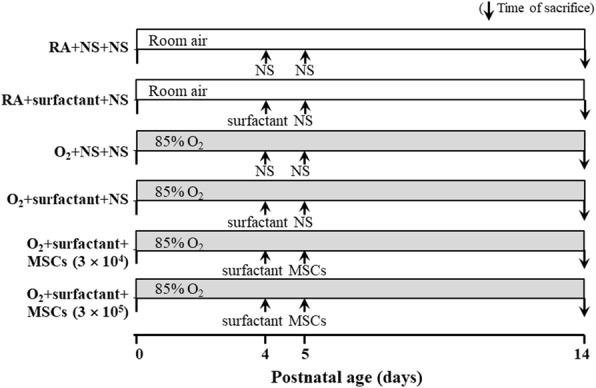
Diagrammatic representation of the experimental design showing the study timeline and the rat pup treatment groups. Neonatal Sprague Dawley rats were reared in either room air (RA) or hyperoxia (85% O_2_) from postnatal days 1 to 14. On postnatal day 4, the rats received intratracheal injections of either 20 μL of normal saline (NS) or 20 μL of surfactant. On postnatal day 5, the rats reared in RA received intratracheal NS, and the rats reared in O_2_ received intratracheal NS or hUC-MSCs (3 × 10^4^ or 3 × 10^5^ cells). MSCs mesenchymal stem cells

This study was approved by the Animal Care Use Committee of Taipei Medical University (LAC-2019-0396). Time-dated pregnant Sprague Dawley rats were housed in individual cages with ad libitum access to laboratory food and water, kept on a 12:12-h light–dark cycle, and allowed to deliver vaginally at term. Within 12 h of birth, the litters were pooled and randomly redistributed to the newly delivered mothers; the pups were then randomly assigned to room air (RA) or oxygen-enriched atmosphere (85% O_2_) groups for postnatal days 1–14. The nursing mothers were rotated between the 85% O_2_ and the RA groups every 24 h to prevent oxygen toxicity in the mothers and to eliminate differing maternal effects between the groups. An oxygen-rich atmosphere was maintained in a transparent 40 × 50 × 60 cm^3^ plexiglass chamber receiving continuous O_2_ at 4 L/min. The oxygen concentration inside the hyperoxic plexiglass chamber was continuously monitored using an oxygen sensor (Coy Laboratory Products, Grass Lake, MI, USA). For intratracheal transplantation, the rats were anesthetized with isoflurane and restrained on a board at a fixed angle as described by Chen et al. [17]. On postnatal day 4, the rats received intratracheal injections of either 20 μL of NS or 20 μL of surfactant (Survanta, AbbVie Inc.), corresponding to approximately 50 mg/kg of phospholipids (Fig. 2). On postnatal day 5, the rats reared in RA were treated with NS and those reared in O_2_ received intratracheal injections of 20 μL of NS, hUC-MSCs (3 × 10^4^ cells), or hUC-MSCs (3 × 10^5^ cells) in 20 μL of NS. Six study groups were examined: RA + NS + NS, RA + surfactant + NS, O_2_ + NS + NS, O_2_ + surfactant + NS, O_2_ + surfactant + hUC-MSCs (3 × 10^4^ cells), and O_2_ + surfactant + hUC-MSCs (3 × 10^5^ cells). On postnatal day 14, the mice were euthanized with isoflurane in a chamber and the lungs were excised for histological, western blot, and cytokine analyses on postnatal day 14. The animals were sacrificed on postnatal day 14 because most studies preserve the rat pups in hyperoxia for up to 14 days postnatal age and the hyperoxia effects reached the peak after postnatal day 14 [1, 18].

### Lung histology

The lungs were placed in 4% paraformaldehyde, washed with phosphate-buffered saline, and then serially dehydrated in increasing concentrations of ethanol before being embedded in paraffin. To standardize the analyses, lung sections were taken from the right middle lobe. Tissue sections at 5-μm thickness were stained with hematoxylin and eosin, examined using light microscopy, and assessed for lung histology. The mean linear intercept (MLI), an indicator of the mean alveolar diameter, was assessed in 10 nonoverlapping fields [14]. Briefly, the number of intercepts is counted on both the horizontal and the vertical field, two numbers per field were obtained, their average calculated and used in the equation: Lm = N × L/m, Lm = MLI, *m* = the sum of all the intercepts, *L* = the length of the traverses, and *N* = the number of times the traverses are placed on the lung.

### Immunohistochemistry of lung vascular endothelial growth factor and von Willebrand factor

Immunohistochemical staining was performed on the 5-μm paraffin sections through immunoperoxidase visualization. After routine deparaffinization, heat-induced epitope retrieval was performed by immersing the slides in 0.01 M sodium citrate buffer (pH 6.0). To block the endogenous peroxidase activity and the nonspecific binding of antibodies, the sections were preincubated for 1 h at room temperature in 0.1 M phosphate-buffered saline containing 10% normal goat serum and 0.3% H_2_O_2_. The sections were then incubated for 20 h at 4 °C with rabbit polyclonal anti–von Willebrand factor (vWF) antibodies (1:100; Abcam, Cambridge, MA, USA) or rabbit polyclonal anti–vascular endothelial growth factor (VEGF) antibodies (1:50; Santa Cruz Biotechnology, Inc., CA, USA) as primary antibodies. The sections were then treated for 1 h at 37 °C with biotinylated goat antimouse or antirabbit IgG (1:200, Jackson ImmunoResesarch Laboratories Inc., West Grove, PA, USA). After the reagents from an avidin–biotin complex kit (Vector Laboratories, Inc., CA, USA) produced a reaction, the reaction products were visualized with a diaminobenzidine substrate kit (Vector Laboratories Inc.) in accordance with the recommendations of the manufacturer. Pulmonary vessel density was determined by counting the number of vessels with positive vWFs stained in an unbiased manner by using a minimum of four random lung fields at × 400 magnification [19].

### Western blot analysis of growth factors

The lung tissues were homogenized in ice-cold buffer containing 50 mM Tris-HCl (pH 7.5), 1 mM EGTA, 1 mM EDTA, and a protease inhibitor cocktail (complete minitablets; Roche, Mannheim, Germany). The samples were sonicated and then centrifuged at 500*g* for 20 min at 4 °C to remove cellular debris. Proteins (30 μg) were resolved on 12% SDS-PAGE gels under reducing conditions and electroblotted to a polyvinylidene fluoride membrane (ImmobilonP, Millipore, Bedford, MA, USA). After blocking with 5% nonfat dry milk, the membranes were incubated with antibodies against VEGF (1:1000; Santa Cruz Biotechnology, Inc.), platelet-derived growth factor subunit B (PDGF-B; 1:1000; Santa Cruz Biotechnology, Inc.), or anti–β-actin (1:20,000; Sigma-Aldrich, St. Louis, MO, USA) and subsequently with horseradish peroxidase-conjugated goat antirabbit IgG or antimouse IgG (Pierce Biotechnology, Rockford, IL, USA). Densitometric analysis was performed with AIDA software to measure the intensity of VEGF, PDGF-B, and β-actin bands.

### Lung cytokine levels

The lung tissue was homogenized in 1 mL of ice-cold lysis buffer containing 1% Nonidet P-40, 0.1% sodium dodecyl sulfate, 0.01 M deoxycholic acid, and a complete protease cocktail inhibitor. Cell extracts were centrifuged, and the levels of interleukin (IL)-1β and IL-6 in the supernatants were measured with an enzyme-linked immunosorbent assay kit (Cloud-Clone Corp., Houston, TX, USA).

### Statistical analysis

Data are presented as box-and-whisker plots. Statistical analyses were performed using one-way ANOVA with the Bonferroni post hoc test for the multiple-group comparisons. The survival rate was evaluated by using the Kaplan–Meier method, and the log-rank test was used for the intergroup comparisons. Differences were considered statistically significant when *P* < 0.05.

## Results

### Surfactant and hUC-MSC treatment increase survival rate

**Fig. 3:**
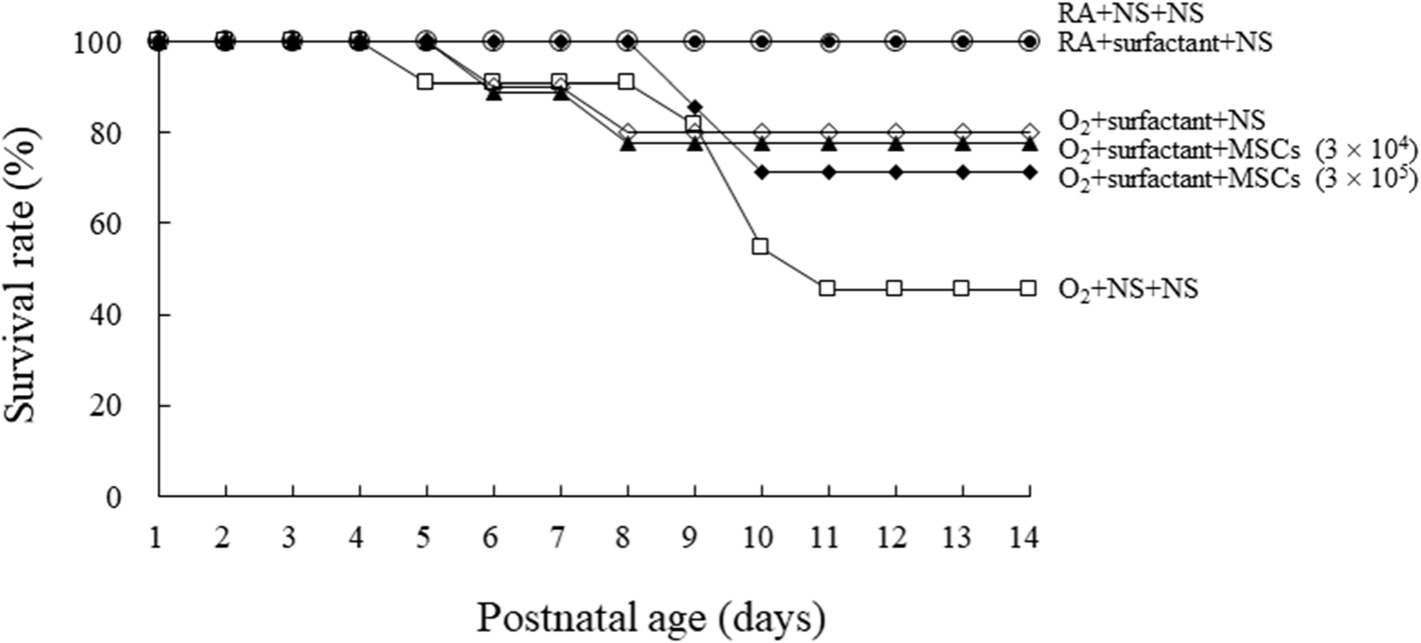
Effects of surfactant and hUC-MSCs on the survival rate on postnatal day 14. All the rats reared in RA and treated with NS or surfactant survived. The rats reared in hyperoxia and treated with NS exhibited a significantly lower survival rate than did those reared in RA and treated with NS or surfactant. Treatment with surfactant and treatment with surfactant and hUC-MSCs augmented the hyperoxia-induced decrease in the survival rate, but the differences in survival rate were not statistically significant. MSCs mesenchymal stem cells, NS normal saline, O_2_ oxygen-enriched atmosphere, RA room air

All the rats reared in RA and treated with NS or surfactant survived (Fig. 3). In the O_2_ + NS + NS group, the death numbers on postnatal days 5 (*n* = 1), 9 (*n* = 1), 10 (*n* = 3), and 11 (*n* = 1). In the O_2_ + surfactant + NS and O_2_ + surfactant + hUC-MSCs (3 × 10^4^ cells) groups, the death numbers on postnatal days 6 (*n* = 1) and 8 (*n* = 1). In the O_2_ + surfactant + hUC-MSCs (3 × 10^5^ cells) groups, the death numbers on postnatal days 9 (*n* = 1) and 10 (*n* = 1). The rats reared in hyperoxia and treated with NS exhibited a significantly lower survival rate than did those reared in RA and treated with NS or surfactant (*P* < 0.05). Treatment with surfactant and treatment with surfactant and hUC-MSCs increased the survival rate compared to untreated rats and the differences were not statistically significant.

### Body and lung weight and lung-to-body-weight ratio

The body and lung weights and the lung-to-body-weight ratios on postnatal day 14 were comparable among the six study groups (Table 1).

**Table 1:** Body and lung weights and lung-to-body-weight ratios in 14-day-old rats exposed to RA or hyperoxia and treated with NS or surfactant on postnatal day 4 and NS or MSCs on postnatal day 5

Treatment	*n*	Body weight	Lung weight	Lung to body weight ratio
		(g)	(g)	(%)
RA + NS + NS	13	23.62 ± 2.57	0.41 ± 0.03	1.74 ± 0.21
RA + surfactant + NS	11	25.75 ± 4.94	0.45 ± 0.06	1.79 ± 0.20
O_2_ + NS + NS	5	23.22 ± 7.01	0.39 ± 0.17	1.64 ± 0.50
O_2_ + surfactant + NS	8	22.83 ± 4.62	0.42 ± 0.09	1.86 ± 0.26
O_2_ + surfactant + hUC-MSCs (3 × 10^4^)	8	24.39 ± 7.07	0.46 ± 0.12	1.94 ± 0.47
O_2_ + surfactant + hUC-MSCs (3 × 10^5^)	5	23.41 ± 1.67	0.40 ± 0.04	1.70 ± 0.13

Values are presented as means ± standard deviations

*hUC-MSCs* human umbilical cord-derived mesenchymal stem cells, *NS* normal saline, *O*_*2*_ oxygen-enriched atmosphere, *RA* room air

### Surfactant and hUC-MSC treatment improve lung development

**Fig. 4:**
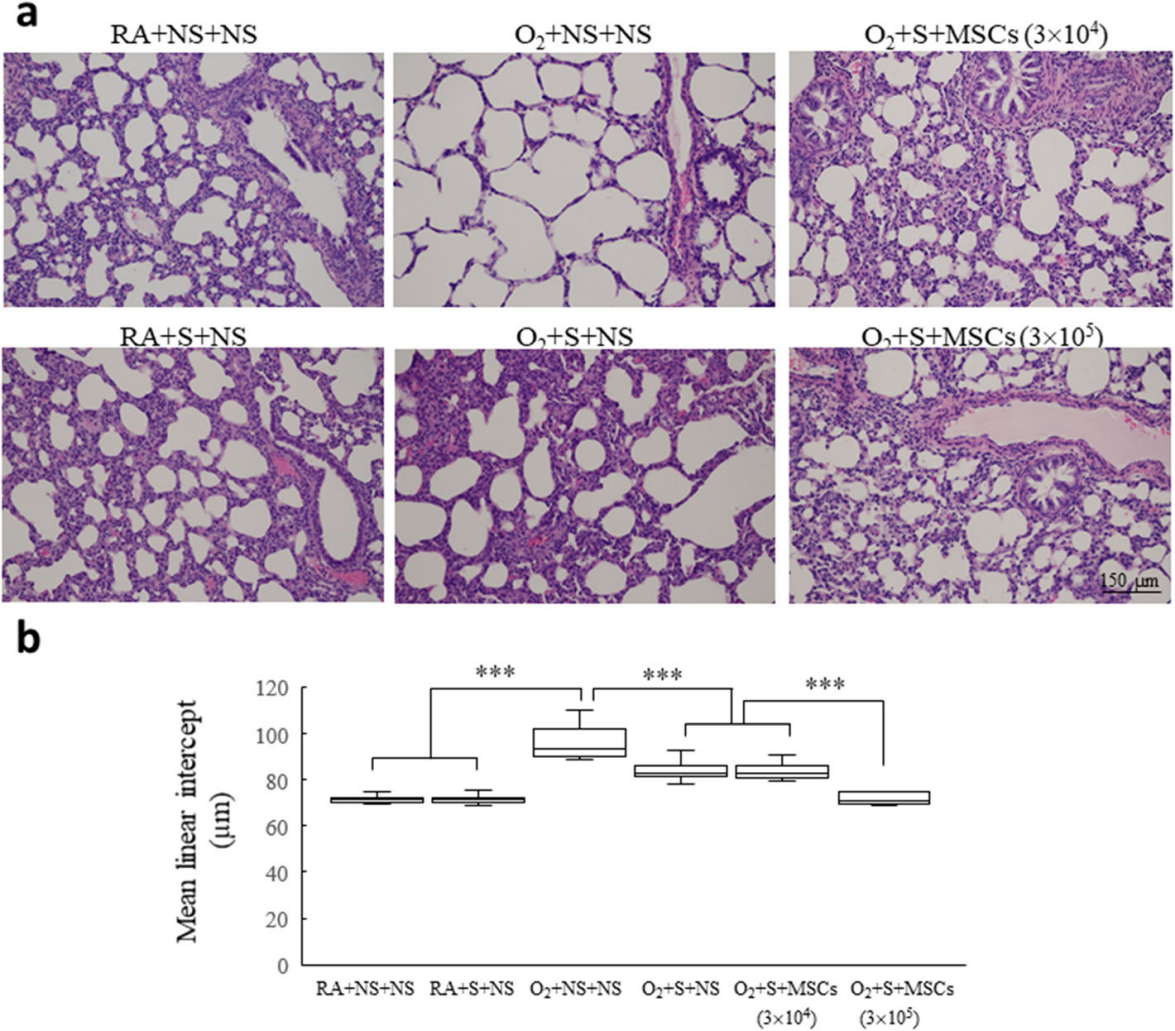
**a** Representative histology and **b** MLI in 14-day-old rats exposed to either postnatal RA or hyperoxia and treated with either NS or surfactant on postnatal day 4 and either NS or hUC-MSCs (3 × 10^4^ or 3 × 10^5^ cells) on postnatal day 5. The rats reared in hyperoxia and treated with NS yielded a significantly higher MLI than did those reared in RA and treated with NS or surfactant. Treatment with surfactant and treatment with surfactant and hUC-MSCs (3 × 10^4^ cells) significantly diminished the hyperoxia-induced increase in the MLI. The O_2_ + surfactant + hUC-MSCs (3 × 10^5^ cells) group exhibited a significantly lower MLI than the O_2_ + surfactant + NS and the O_2_ + surfactant + hUC-MSCs (3 × 10^4^ cells) groups. Data are shown as box-and-whisker plots. ***P < 0.001. MSCs mesenchymal stem cells, NS normal saline, O_2_ oxygen-enriched atmosphere, RA room air, S surfactant

**Fig. 5:**
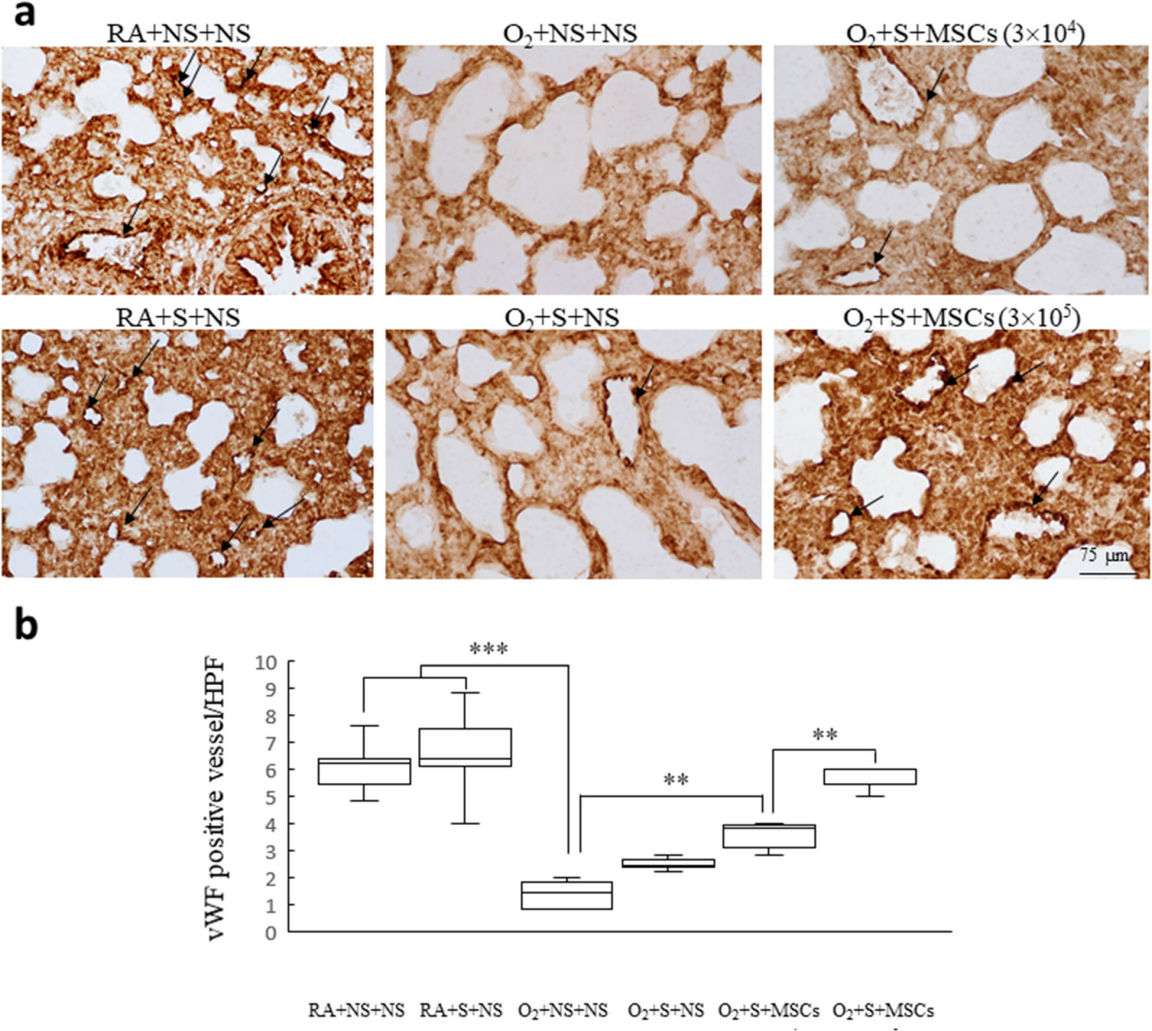
**a** Representative histology and **b** vascular density in 14-day-old rats exposed to either postnatal RA or hyperoxia and treated with either NS or surfactant on postnatal day 4 and either NS or hUC-MSCs (3 × 10^4^ or 3 × 10^5^ cells) on postnatal day 5. The endothelium of the blood vessel with vWF immunoreactivity was indicated by the black arrow. The rats reared in hyperoxia and treated with NS yielded a significantly lower vascular density than those reared in RA and treated with NS or surfactant. Treatment with surfactant and hUC-MSCs (3 × 10^4^ or 3 × 10^5^ cells) significantly augmented the hyperoxia-induced decrease in the vascular density. Treatment with surfactant and hUC-MSCs (3 × 10^5^ cells) more significantly increased vascular density than did the treatment of surfactant and hUC-MSCs (3 × 10^4^ cells). Data are shown as box-and-whisker plots. **P < 0.01 and ***P < 0.001. MSCs mesenchymal stem cells, NS normal saline, O_2_ oxygen-enriched atmosphere, RA room air, S surfactant, vWF von Willebrand factor

Figure 4 presents the lung tissue sections stained with hematoxylin and eosin on postnatal day 14. The rats reared in hyperoxia and treated with NS exhibited large thin-walled air spaces and yielded a significantly higher MLI than did those reared in RA and treated with NS or surfactant (Fig. 4a). Treatment with surfactant and treatment with surfactant and hUC-MSCs (3 × 10^4^ cells) significantly diminished the hyperoxia-induced increase in the MLI. The O_2_ + surfactant + hUC-MSCs (3 × 10^5^ cells) group exhibited a significantly lower MLI than did the O_2_ + surfactant + NS and the O_2_ + surfactant + hUC-MSCs (3 × 10^4^ cells) groups (Fig. 4b). Figure 5 shows representative lung sections stained for vWF on postnatal day 14. The rats reared in hyperoxia and treated with NS yielded a significantly lower vascular density than did those reared in RA and treated with NS or surfactant. Treatment with surfactant and hUC-MSCs (3 × 10^4^ or 3 × 10^5^ cells) significantly augmented the hyperoxia-induced decrease in vascular density. The surfactant + hUC-MSCs (3 × 10^5^ cells) treatment more significantly increased vascular density compared with the surfactant + hUC-MSCs (3 × 10^4^ cells) treatment.

### Surfactant and hUC-MSC treatment increase VEGF expression

**Fig. 6:**
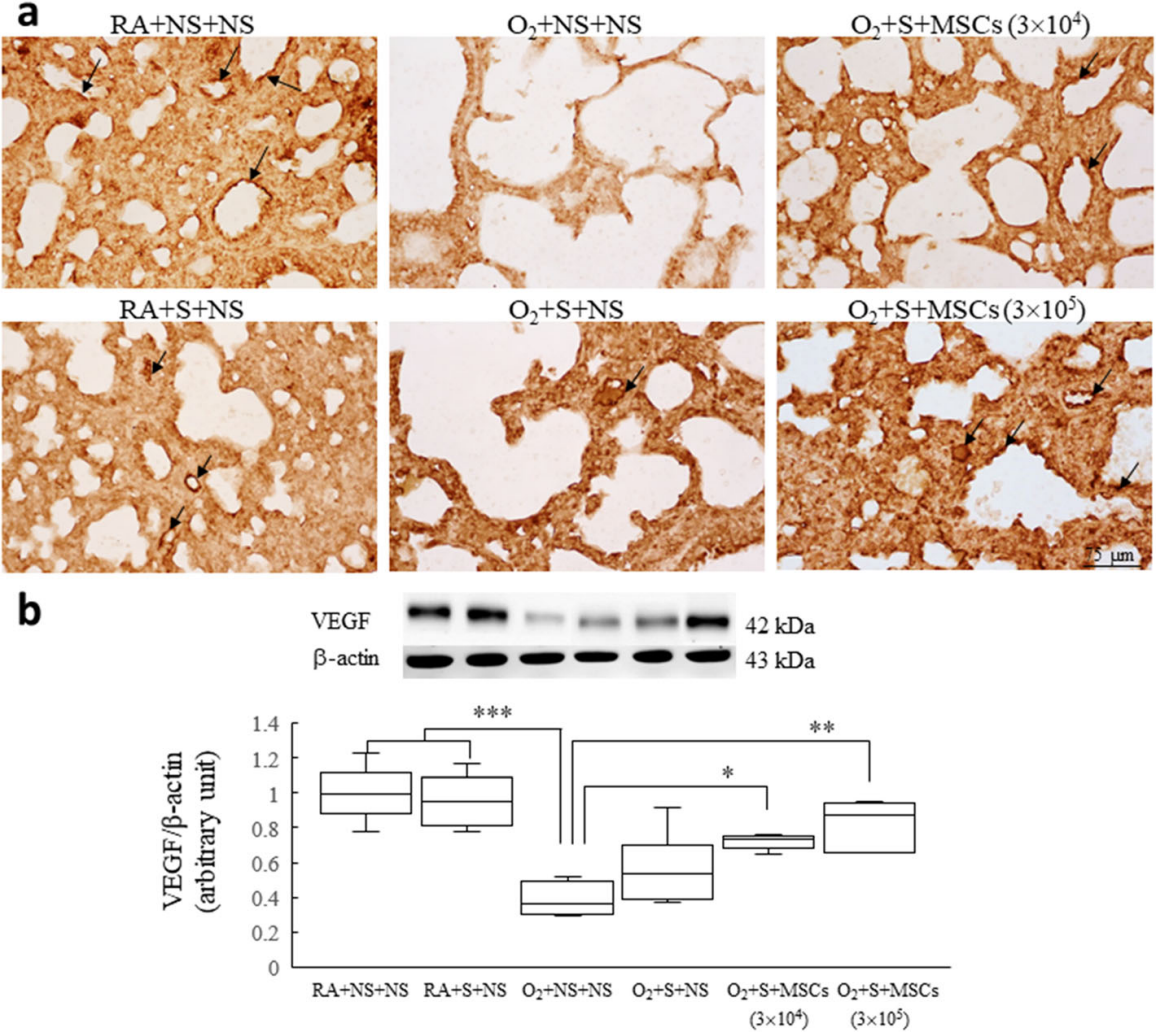
**a** Representative immunohistochemistry of VEGF and **b** representative western blots and quantitative data determined with densitometry for VEGF protein expression in 14-day-old rats exposed to postnatal RA or hyperoxia and treated with NS or surfactant on postnatal day 4 and NS or hUC-MSCs (3 × 10^4^ or 3 × 10^5^ cells) on postnatal day 5. The black arrows indicated the VEGF positively stained endothelium of the blood vessel. The rats reared in hyperoxia and treated with NS exhibited significantly lower levels of VEGF protein expression than did those reared in RA and treated with NS or surfactant. Treatment with surfactant and hUC-MSCs (3 × 10^4^ or 3 × 10^5^ cells) more significantly augmented the hyperoxia-induced decrease in the VEGF protein expression levels than the treatment with NS. Data are shown as box-and-whisker plots. *P < 0.05, **P < 0.01, and ***P < 0.001. MSCs mesenchymal stem cells, NS normal saline, O_2_ oxygen-enriched atmosphere, RA room air, S surfactant, VEGF vascular endothelial growth factor

The VEGF immunoreactivities were primarily detected in the endothelial cells of blood vessels (Fig. 6a). The rats reared in hyperoxia and treated with NS exhibited significantly lower VEGF protein expression than did those reared in RA and treated with NS or surfactant (Fig. 6b). Treatment with surfactant and hUC-MSCs (3 × 10^4^ or 3 × 10^5^ cells) significantly augmented the hyperoxia-induced decrease in VEGF protein expression compared with treatment with NS.

### Surfactant and hUC-MSC treatment increase PDGF expression

**Fig. 7:**
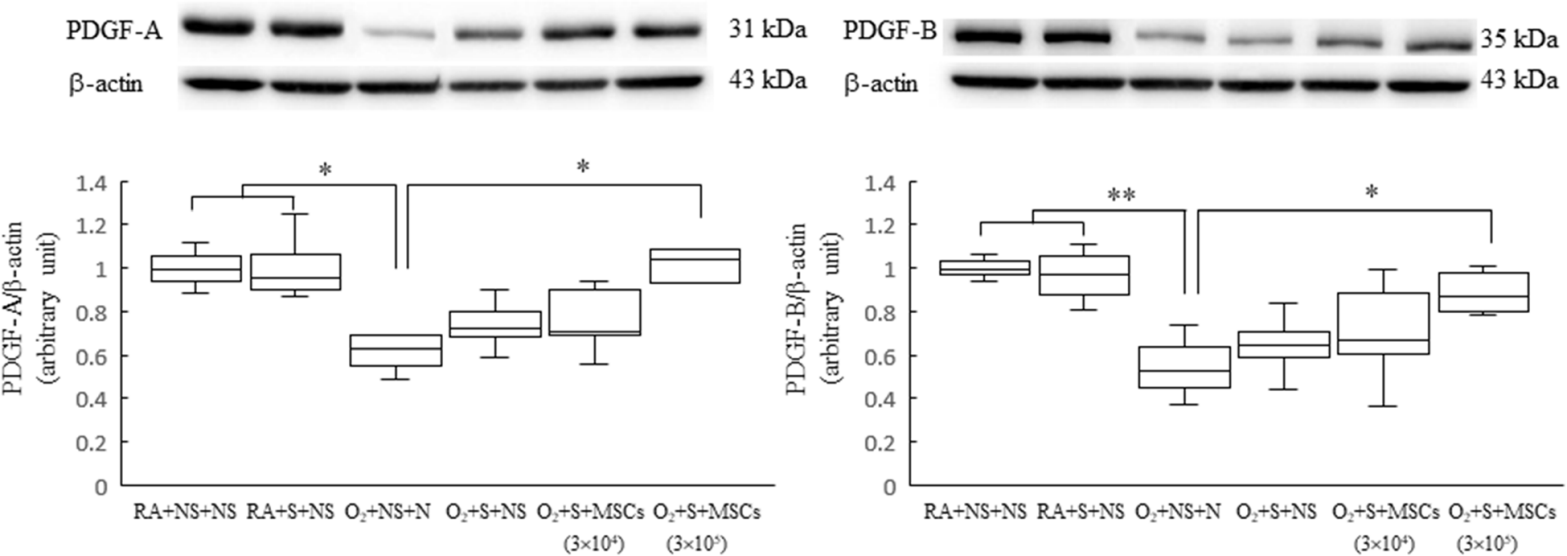
Representative western blots and quantitative data determined with densitometry for PDGF-A and PDGF-B protein expression in 14-day-old rats exposed to postnatal RA or hyperoxia and treated with NS or surfactant on postnatal day 4 and NS or MSCs (3 × 10^4^ or 3 × 10^5^ cells) on postnatal day 5. The rats reared in hyperoxia and treated with NS exhibited significantly lower PDGF-A and PDGF-B protein expression levels than those reared in RA and treated with NS or surfactant. Treatment with surfactant and hUC-MSCs (3 × 10^5^ cells) more significantly augmented the hyperoxia-induced decrease in the PDGF-A and PDGF-B protein expression levels than did the treatment with NS. Data are shown as box-and-whisker plots. *P < 0.05 and **P < 0.01. MSCs mesenchymal stem cells, NS normal saline, O_2_ oxygen-enriched atmosphere, PDGF platelet-derived growth factor, RA room air, S surfactant

Figure 7 shows the representative western blot of PDGF-A and PDGF-B. The rats reared in hyperoxia and treated with NS exhibited significantly lower PDGF-A and PDGF-B protein expression than did those reared in RA and treated with NS. Treatment with surfactant and hUC-MSCs (3 × 10^5^ cells) significantly augmented the hyperoxia-induced decrease in the PDGF-A and PDGF-B protein expression compared with treatment with NS.

### Surfactant and hUC-MSC treatment decrease lung cytokine levels

**Fig. 8:**
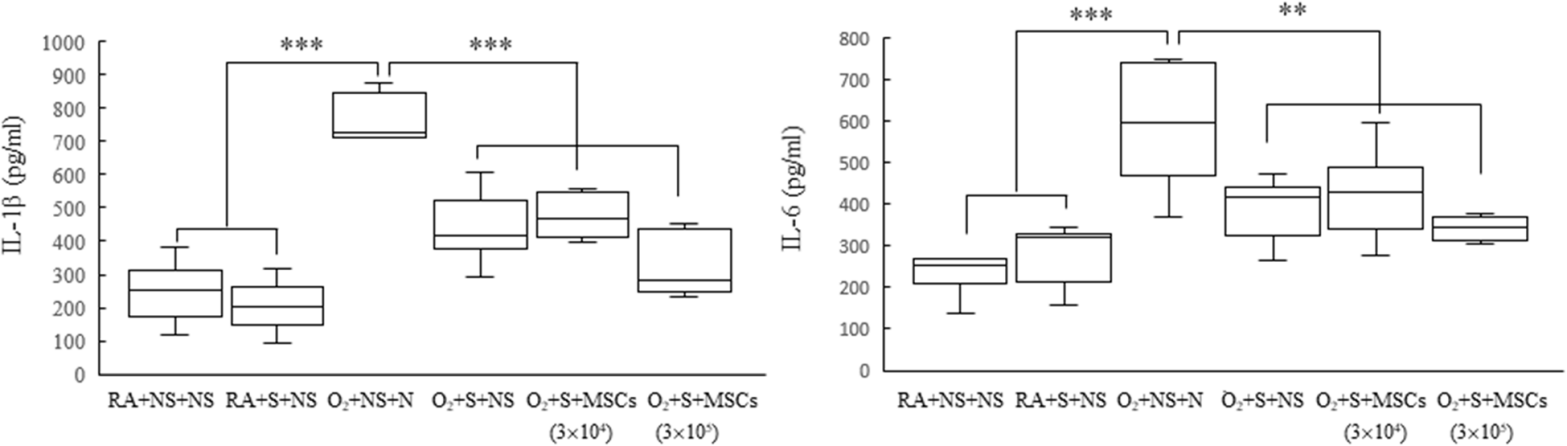
Lung IL-1β and IL-6 levels in 14-day-old rats exposed to postnatal RA or hyperoxia and treated with NS or surfactant on postnatal day 4 and NS or hUC-MSCs (3 × 10^4^ cells or 3 × 10^5^ cells) on postnatal day 5. The rats reared in hyperoxia and treated with NS yielded a significantly higher IL-1β and IL-6 levels than did those reared in RA and treated with NS or surfactant. Treatment with surfactant and surfactant + hUC-MSCs (3 × 10^4^ or 3 × 10^5^ cells) significantly reduced the hyperoxia-induced increase in the IL-1β and IL-6 levels. Data are shown as box-and-whisker plots. **P < 0.01 and ***P < 0.001. S: surfactant. MSCs mesenchymal stem cells, NS normal saline, O_2_ oxygen-enriched atmosphere, RA room air, S surfactant

The rats reared in hyperoxia and treated with NS yielded significantly higher IL-1β and IL-6 levels than did those reared in RA and treated with NS or surfactant (Fig. 8). The treatment with surfactant and the treatment with surfactant and hUC-MSCs significantly diminished the hyperoxia-induced increase in IL-1β and IL-6 levels.

## Discussion

The main findings of our in vivo neonatal rat model were that the intratracheal administration of hUC-MSCs on the day following administration of surfactant improves lung development and that high doses of hUC-MSCs amplify the therapeutic effects on experimental BPD in neonatal rats compared with the low doses of hUC-MSCs.

Although term-born rats have structurally immature lungs, they are functionally mature and require no surfactant treatment. In one study, neonatal mice exposed to hyperoxia for 4 days exhibited disruptions to type II cell proliferation, which produced pulmonary surfactant [20]. In another study, hyperoxia during the first 3 days of life induced inflammatory cell infiltration in alveolar spaces and increased the wet-to-dry lung weight ratio in neonatal Sprague Dawley rats [21]. The results of these studies have suggested that hyperoxia reduces surfactant production and induces lung inflammation in newborn animals. Pulmonary surfactant is a mixture of phospholipids, surfactant-associated proteins, and neutral lipids, which modulate pulmonary inflammation and stabilize the alveoli by reducing surface tension [22]. In our study, the administration of surfactant on postnatal day 4 diminished the hyperoxia-induced increase in MLI and lung cytokines in the neonatal rats. The surfactant treatment did not augment the hyperoxia-induced decrease in pulmonary vascular density. These results support the idea that pulmonary surfactant fulfills an essential role in the lungs for both host defense mechanisms, such as modulating pulmonary inflammation, and for improving alveolarization [14, 23].

Surfactant therapy has become the standard of care for preterm infants with respiratory distress syndrome and can reduce the combined outcomes of death and BPD [6]. Compared with delayed surfactant treatment, early surfactant treatment was more effective in reducing mortality, air leak, BPD, and BPD or death in preterm infants [15]. Although surfactant has a unique spreading property and can reduce surface tension. The addition of a surfactant reduced the in vitro viability of hUC-MSCs, and the combination therapy of surfactant and hUC-MSCs did not exhibit any additional benefits to lung development in neonatal rats exposed to hyperoxia [14]. The therapeutic effect of MSCs in ameliorating the hyperoxia-induced lung injury has been reported in our previous studies [13, 14]. For this reason, we administered intratracheal surfactant and hUC-MSCs on 2 consecutive days and found that the intratracheal administration of surfactant on postnatal day 4 and hUC-MSCs on postnatal day 5 improved alveolarization and angiogenesis in the neonatal rats exposed to hyperoxia. The time interval between the administration of surfactant and the hUC-MSCs for achieving optimal therapeutic effects was not determined. Future studies are required to evaluate the effects of different time intervals on hyperoxia-induced lung injury.

In this study, the administration of surfactant alone and the administration of surfactant with hUC-MSCs to the hyperoxia-exposed rats improved lung development in the surviving animals, although the survival rate did not significantly improve. The differences in the survival rates between rats treated with surfactant and those treated with surfactant and hUC-MSCs were not significant on postnatal day 14. The rats reared in hyperoxia and treated with NS exhibited a low survival rate after postnatal day 5. The treatment with surfactant alone and treatment with surfactant and hUC-MSCs did not improve the survival rate. These results suggest that an additional dose of hUC-MSCs is required to maintain the survival rate.

In this study, we determined the levels of VEGF, PDGF-A, and PDGF-B expression and elucidated the mechanisms that mediate the hUC-MSCs’ effects because their mRNA and protein expression decreased in the lungs of newborn animals exposed to 14 days of hyperoxia [3, 24, 25]. VEGF is a potent endothelial cell mitogen that regulates angiogenesis and alveolar development [26]. PDGF is crucial to the alveolarization of normally developing lungs [27]. We demonstrated that the rats reared in hyperoxia and treated with NS exhibited significantly lower levels of VEGF, PDGF-A, and PDGF-B protein expression than did those reared in RA and treated with NS or surfactant. Treatment with surfactant and hUC-MSCs augmented the hyperoxia-induced decrease in the VEGF, PDGF-A, and PDGF-B protein expression levels. These results suggest that treatment with hUC-MSCs enhanced vascular and alveolar development in the neonatal rats through the induction of growth factors.

Prolonged exposure to hyperoxia increases cytokine and induces inflammation in neonatal rat lungs [3]. Oxygen supplementation in preterm infants with respiratory distress syndrome increases oxidative stress and cytokines and the cytokine levels were increased in the tracheal aspirate of newborns with BPD [28]. These results indicate that cytokines play a crucial role in the development of BPD [29]. MSCs have immunomodulatory and anti-inflammatory effects and have been reported to reduce a neonatal hyperoxia-induced increase in IL-6 levels [12]. In this study, the rats reared in hyperoxia and treated with NS exhibited a significant increase in IL-1β and IL-6 levels, and these were decreased with the treatment of surfactant and hUC-MSCs. These results support previous studies and suggest that the therapeutic effects of surfactant and hUC-MSCs on hyperoxia-induced lung injury are mediated through the inhibition of proinflammatory cytokine production [12, 13].

Our study has several limitations. First, we did not evaluate the effects of surfactant and MSCs on pulmonary hypertension. Pulmonary hypertension is reported in one third of extremely low birth weight infants with severe BPD and is associated with considerable mortality and morbidity [30, 31]. Preclinical studies have demonstrated the efficacy of MSCs in alleviating hyperoxia-induced BPD and pulmonary hypertension [32, 33]. Second, we did not examine the collagen and elastin expression. Previous study demonstrated that hyperoxia disturbed collagen and elastin cross-linking, impacted lung rigidity and elasticity, and arrested lung development in an animal model of BPD [34]. Additional studies are warranted to evaluate the effects of surfactant and MSCs on pulmonary hypertension and collagen to elastin ratio in hyperoxia-induced lung injury.

## Conclusions

Consecutive daily administration of intratracheal surfactant and hUC-MSCs likely attenuated hyperoxia-induced defective alveolarization and angiogenesis by increasing VEGF expression. High doses of hUC-MSCs enhanced the therapeutic effects more effectively than the low doses of hUC-MSCs. Consecutive daily administration of intratracheal surfactant and hUC-MSCs can be an effective regimen for treating hyperoxia-induced lung injury in neonates.

## Data Availability

Not applicable.

## Abbreviations

BPD: Bronchopulmonary dysplasia
hUC-MSCs: Human umbilical cord-derived mesenchymal stem cells
IL: Interleukin
MLI: Mean linear intercept
NS: Normal saline
O_2_: Oxygen-enriched atmosphere
PDGF: Platelet-derived growth factor
RA: Room air
VEGF: Vascular endothelial growth factor
vWF: von Willebrand factor
